# Association of early-life factors with prematurity-associated lung disease: prospective cohort study

**DOI:** 10.1183/13993003.01766-2021

**Published:** 2022-05-12

**Authors:** Kylie Hart, Michael Cousins, W. John Watkins, Sarah J. Kotecha, A. John Henderson, Sailesh Kotecha

**Affiliations:** 1 Dept of Child Health, Cardiff University School of Medicine, Cardiff, UK; 2 Neonatal Unit, Cardiff and Vale University Health Board, Cardiff, UK; 3 MRC Integrative Epidemiology Unit, Population Health Sciences, Bristol Medical School, University of Bristol, Bristol, UK

## Abstract

**Background:**

Although bronchopulmonary dysplasia (BPD) is associated with lung function deficits in childhood, many who develop BPD have normal lung function in childhood and many without BPD, including those born at 33–34 weeks of gestation, have lung dysfunction in childhood. Since the predictability of BPD for future lung deficits is increasingly doubted, we prospectively recruited preterm-born children to identify early-life factors associated with lung function deficits after preterm birth.

**Methods:**

From 767 children aged 7–12 years who had their respiratory symptoms assessed, and had spirometry before and after a bronchodilator in our Respiratory Health Outcomes in Neonates (RHiNO) study, 739 (544 preterm-born at ≤34 weeks of gestation and 195 term-born) had satisfactory lung function. Data were analysed using multivariable logistic regression and mediation.

**Results:**

When preterm-born children were classified according to their lung function, low lung function (prematurity-associated lung disease (PLD)) was associated with BPD, gestation and intra-uterine growth restriction (IUGR) on univariable logistic regression analyses. However, on multivariable logistic regression analyses, gestation (β= –0.153, se 0.051; p=0.003) and IUGR (OR 1.783, 95% CI 1.06–3.00; p=0.029) remained significantly associated with later deficits of lung function, but BPD (OR 0.99, 95% CI 0.52–1.89; p=0.974) did not. Mediation analyses confirmed these results.

**Conclusions:**

Although traditionally BPD has been associated with low lung function in later life, the data show that gestation and IUGR are significantly associated with PLD in childhood, but BPD is not. By identifying children with PLD, we can better understand the underlying mechanisms and develop optimal therapies.

## Abstract 2

**Although traditionally bronchopulmonary dysplasia is thought to be associated with longer term lung function deficits, this study shows that gestation and fetal growth restriction are better predictors of lung function deficits in prematurely born children** https://bit.ly/3obSdSz

## Introduction

It is now well established that prematurity is associated with long-term respiratory deficits in childhood and young adulthood [1–5]. These deficits are particularly marked in those who develop bronchopulmonary dysplasia (BPD; often also called chronic lung disease of prematurity) in infancy [6–8], but even those who are born at 33–34 weeks of gestation [9–12] are also at significant risk of developing respiratory disease in later life. Preterm-born infants are delivered at an early stage of lung development and may be exposed to antenatal (chorioamnionitis, maternal disease or placental dysfunction) or postnatal (mechanical ventilation, supplemental oxygen or infection) risk factors of lung injury; thus, lung disease may result from these different exposures [13, 14]. As summarised by our earlier systematic review [1], most individual studies reported decreased spirometry after preterm birth, including those who did and did not develop BPD, but in general were small in size. Therefore, the studies did not permit detailed analyses of which early-life factors may be important in the development of future lung function deficits. Since BPD is increasingly questioned for predicting future lung function deficits [15, 16], it is important to assess which early-life factors, including gestation, BPD, intra-uterine growth restriction (IUGR), sex, *etc.*, may be important in these future lung function deficits in preterm-born children.

We prospectively studied the largest population to date of children aged 7–12 years who were born at ≤34 weeks of gestation to specifically identify those with low spirometry to permit identification of which early-life factors are associated with lung function deficits associated with preterm birth.

## Methods

In 2013, we invited all surviving preterm-born children together with matched term-born children who were born throughout Wales, then aged 1–3, 5, 7 and 9 years, *via* a respiratory questionnaire [3, 17]. We re-invited the responders and the nonresponders from this 2013 cohort, adding additional potential participants who were born in South Wales (to permit accessibility) from the NHS Wales Informatics Service if they were aged 7–12 years at the time of mailing the invitations. Thus, those born and cared for at all levels of neonatal care were eligible for inclusion. We mailed invitations between November 2016 and September 2019 to join the Respiratory Health Outcomes in Neonates study (RHiNO; EudraCT: 2015-003712-20) for comprehensive assessments if they were aged 7–12 years, born at ≤34 weeks of gestation for the preterm group or at ≥37 weeks of gestation for the control group. Ethical approval was obtained from the South-West Bristol Research Ethics Committee (15/SW/0289). For parents and children who provided informed written consent and assent, respectively, two research nurses evaluated the children at their home or in hospital. After checking the accuracy of the parent-completed respiratory questionnaires and conducting a physical examination, the research nurses measured the children's height, weight, exhaled nitric oxide fraction (*F*_ENO_) (NIOX VERO; Circassia, Oxford, UK) and spirometry (Microloop; CareFusion, Wokingham, UK) before and 15–20 min after administration of a bronchodilator (4×100 μg puffs of salbutamol administered *via* a spacer device) to assess reversibility (increase of forced expiratory volume in 1 s (FEV_1_) >10% predicted). Spirometry was quality controlled as per European Respiratory Society/American Thoracic Society guidelines including independent quality control [18] and normalised against Global Lung Function Initiative reference values [19]. IUGR was defined as <10th centile for birthweight adjusted for sex and gestation using LMSgrowth (Medical Research Council) [20]. Deprivation scores and quintiles were estimated from the participants’ postcodes using the Welsh Index of Multiple Deprivation (WIMD) score, which is a measure of deprivation based on eight domains including wealth, schooling and home ownership [21]. Children with congenital malformations, significant cardiopulmonary disorder or inability to perform spirometry due to severe neurodevelopmental disorders were excluded. All children withheld their drugs prior to their assessments (short- and long-acting β_2_-agonists for 8 and 48 h, respectively, inhaled corticosteroids for 24 h, and leukotriene receptor antagonists for 48 h) [18, 22] and were free of respiratory infections for at least 3 weeks prior to testing. Diagnosis of BPD was based on oxygen requirement at 28 days of age or at 36 weeks post-conceptual age. Information on early-life factors including gestational age, birthweight, diagnosis of BPD (according to the National Institute of Child Health and Human Development criteria using need for supplemental oxygen at 28 days of age and at 36 weeks of corrected gestation for the group born at <32 weeks of gestation at birth; and at 28 and 56 days of age for those born at ≥32 weeks at birth to classify the children into no BPD or BPD including mild, moderate or severe BPD; or into moderate/severe BPD as used in the sensitivity analyses [23]), *etc.*, was obtained from the neonatal medical notes.

### Statistical methods

The mean (95% confidence interval) or median (range) is presented as appropriate. Continuous data were analysed using the independent t-test or Mann–Whitney U-test for two groups and one-way ANOVA or the Kruskal–Wallis test for multiple group comparisons with *post hoc* Bonferroni correction. Pearson's Chi-squared test was used to analyse categorical data. Univariable and multivariable logistic regression modelling was performed to identify potential predictors of low spirometry in preterm-born children. Mediation analyses were performed using Mplus (Muthén & Muthén, Los Angeles, CA, USA); all other analyses used SPSS version 23.0 (IBM, Armonk, NY, USA). Minor differences in data values are due to the slightly different algorithms used. p-values <0.05 were considered statistically significant.

### Power calculation

We had estimated that the power of the multiple regression model using an α of 0.05 and one variable for each 100 participants recruited (estimated to five to six variables) with an R^2^-value of 0.2 to be conservative. There were no controlled independent variables included in the calculation, as this model was being created from scratch with no variables automatically included. This model resulted in a power of 100% and so we had sufficient power if all of the variables were retained in the model.

## Results

The CONSORT diagram shows the details of the invitees and responders (supplementary figure S1). From 1122 (827 preterm, 295 term) responders, 767 (565 preterm, 202 term) were comprehensively assessed, with satisfactory spirometry data available from 544 preterm-born and 195 term-born children. When the responders and nonresponders were compared, there were small differences for gestational age and birthweight, and fewer of the most deprived population responded (supplementary table S1). To identify which early risk factors were associated with low lung function observed after preterm birth, we classified preterm-born children into those with FEV_1_ ≤85% predicted (PT_low_) and >85% predicted (control; PT_c_) groups (table 1). The PT_low_ group had lower birthweight and gestation, but had greater rates of IUGR, BPD, neonatal complications and greater respiratory symptoms in childhood when compared with the PT_c_ and term-born control (T_c_) groups. The PT_low_ group had neither greater family history of atopy nor greater exposure to antenatal or postnatal smoking when compared with the PT_c_ group. Interestingly, although a greater proportion of the preterm-born children who had BPD in infancy (41 out of 108 (38%)) had low spirometry, a significant proportion of the preterm subjects without BPD (100 out of 436 (23%)) also had low spirometry. When the analysis was restricted to those born at ≤32 weeks of gestation, 38% of the BPD group and 25% of the PT_c_ group had low lung function. As expected, the PT_low_ group had low spirometry values as they were classified using low FEV_1_ % pred, but a greater proportion of the PT_low_ group had *F*_ENO_ >35 ppb (28 out of 124 (23%)) when compared with the PT_c_ (33 out of 350 (9%)) or T_c_ (20 out of 183 (11%)) groups (table 2). Similarly, the PT_low_ group had greater bronchodilator responses with an increase in FEV_1_ % pred of 7.9% compared with 4.4% and 3.6% for the PT_c_ and T_c_ groups, respectively, after administration of salbutamol. Furthermore, a greater proportion had a positive bronchodilator response in the PT_low_ group (39 out of 134 (29%)) when compared with the control groups (PT_c_: 27 out of 382 (7%); T_c_: 10 out of 183 (6%)).

**TABLE 1:** Characteristics of participants based upon forced expiratory volume in 1 s (FEV_1_) % predicted

	**Preterm-born group: FEV_1_ ≤85% pred (PT_low_)**	**Preterm-born control group: FEV_1_ >85% pred (PT_c_)**	**Term-born control group (T_c_)**
**Subjects**	141 (26)^#^	403 (74)^#^	195 (26)^¶^
**Current status**			
Male	68/141 (52)	211/403 (52)	100/195 (51)
Current age (years)	9.9 (9.7–10.2)**	9.5 (9.4–9.7)	9.7 (9.5–9.8)
Current height (cm)	140.7 (139.1–142.4)	139.5 (138.4–140.5)^‡^	142.0 (140.7–143.3)
Current weight (kg) (range)	32.4 (18.2–62.8)	32.8 (17.7–88.9)^‡^	34.8 (21.4–78.2)
Current BMI (kg·m^−2^) (range)	16.5 (12.2–28.0)	17.0 (12.8–32.5)	17.1 (13.2–30.9)
**Neonatal history**			
Gestational age (weeks) (range)	31 (24–34)**^,†††^	32 (23–34)^‡‡‡^	40 (37–42)
Birthweight (g) (range)	1450 (482–2930)**^,†††^	1758 (450–3912)^‡‡‡^	3430 (2155–4916)
Birthweight (z-score after adjustments for gestation and sex)	−0.087 (−0.312–0.139)*	0.256 (0.124–0.387)	0.062 (−0.076–0.199)
IUGR	28/141 (20)*^,†††^	48/403 (12)^‡‡^	9/195 (5)
Caesarean section	79/141 (56)^†††^	227/401 (57)^‡‡‡^	49/195 (25)
Antenatal steroids	122/135 (90)^†††^	329/376 (88)^‡‡‡^	4/195 (2)
Postnatal steroids	6/131 (5)^††^	8/383 (2)^‡^	0/194 (0)
BPD	41/141 (29)**	67/403 (16)	0/195
ROP	13/141 (9)^†††^	19/403 (5)^‡‡‡^	0/195 (0)
IVH	26/141 (18)***^,†††^	30/403 (7)^‡‡‡^	0/195 (0)
NEC	13/137 (10)^†††^	18/390 (4)^‡‡^	0/194 (0)
PDA	14/138 (10)^†††^	21/388 (5)^‡‡‡^	0/195 (0)
Combined illness (ROP, IVH or NEC)	41/139 (30)***^,†††^	53/391 (14)^‡‡‡^	0/194 (0)
**Family history**			
Maternal antenatal smoking	14/138 (10)	48/394 (12)^‡‡^	11/194 (6)
Maternal postnatal smoking	16/140 (11)^†^	58/399 (15)^‡‡‡^	8/195 (4)
Family history of asthma	83/140 (59)^†^	213/399 (53)	91/195 (47)
Family history hay fever	72/138 (52)	217/396 (55)	115/194 (59)
Family history eczema	59/138 (43)	183/393 (47)	90/195 (46)
Family history allergies	52/138 (38)	156/393 (40)	81/193 (42)
**Respiratory symptoms**			
Bronchiolitis	39/141 (28)**^,†††^	58/401 (15)^‡‡‡^	10/194 (5)
Wheeze ever	87/138 (63)*^,†††^	194/385 (50)	51/191 (27)
Wheeze last 12 months	48/141 (34)^†††^	104/403 (26)^‡‡‡^	25/195 (13)
Inhalers last 12 months	32/141 (23)^†††^	61/403 (15)^‡‡^	12/195 (6)
Diagnosed asthma	35/140 (25)**^,†††^	53/403 (13)^‡‡^	10/193 (5)

Data are presented as n or n/N with % or 95% CI shown in brackets, unless otherwise stated. BMI: body mass index; IUGR: intra-uterine growth restriction; BPD: bronchopulmonary dysplasia (mild and moderate/severe); ROP: retinopathy of prematurity (all grades); IVH: intraventricular/cerebral haemorrhage (all grades); NEC: necrotising enterocolitis (requiring medical or surgical treatment); PDA: patent ductus arteriosus (requiring medical or surgical treatment). ^#^: % of preterm population; ^¶^: % of total population. PT_c_ *versus* T_c_: ^‡^: p<0.05; ^‡‡^: p<0.01; ^‡‡‡^: p<0.001. PT_low_ *versus* PT_c_: *: p<0.05; **: p<0.01; ***: p<0.001. PT_low_ *versus* T_c_: ^†^: p<0.05; ^††^: p<0.01; ^†††^: p<0.001.

**TABLE 2:** Spirometry, reversibility and exhaled nitric oxide fraction (*F*_ENO_) for the term (T_c_) and preterm population classified into low (PT_low_) and normal (PT_c_) lung function

	**Preterm-born group: FEV_1_ ≤85% pred (PT_low_)**	**Preterm-born control group: FEV_1_ >85% pred (PT_c_)**	**Term-born control group (T_c_)**
**Baseline spirometry**	n=141	n=403	n=195
FEV_1_ % pred	75.6 (74.0–77.1)^†††,^***	96.6 (95.8–97.4)	95.7 (94.2–97.0)
FVC % pred	83.2 (81.8–84.6)^†††,^***	98.2 (97.2–99.1)	96.2 (94.8–97.7)
FEV_1_/FVC	0.80 (0.78–0.81)^†††,^***	0.86 (0.86–0.87)	0.87 (0.86–0.88)
FEF_25–75%_ % pred	57.0 (54.2–59.7)^†††,^***	84.0 (82.3–85.6)	86.4 (83.6–89.1)
**Post-BD spirometry**	n=134	n=382	n=183
FEV_1_ % pred	83.3 (81.8–84.9)^†††,^***	101.0 (100.1–102.0)	99.0 (97.5–100.5)
FVC % pred	86.4 (84.7–88.2)^†††,^***	99.0 (98.0–100.0)^‡^	96.7 (95.2–98.3)
FEV_1_/FVC	0.85 (0.83–0.86)^†††,^***	0.89 (0.89–0.90)	0.89 (0.89–0.90)
FEF_25–75%_ % pred	71.8 (68.8–74.8)^†††,^***	97.0 (95.3–98.8)	96.2 (93.4–99.1)
**Mean change in pre-/post-BD spirometry**	n=134	n=382	n=183
FEV_1_ % pred	7.9 (6.5–9.3)^†††,^***	4.4 (3.9–4.8)	3.6 (2.9–4.3)
FVC % pred	3.0 (1.8–4.2)^†††,^***	0.6 (0.1–1.0)	0.5 (−0.1–1.1)
FEV_1_/FVC	0.054 (0.044–0.064)^†††,^***	0.032 (0.028–0.036)	0.029 (0.023–0.035)
FEF_25–75%_ % pred	15.5 (13.6–17.4)^††^	13.3 (12.1–14.6)	11.0 (8.9–13.0)
**Positive BD response**	39 (29)^†††,^***	27 (7)	10 (6)
***F*_ENO_ >35 ppb**	n=124	n=350	n=183
	28 (23)^††,^***	33 (9)	20 (11)

Data are presented as n with 95% CI or % shown in brackets. FEV_1_: forced expiratory volume in 1 s; FVC: forced vital capacity; FEF_25–75%_: forced expiratory flow at 25–75% of FVC; BD: bronchodilator. PT_c_ *versus* T_c_: ^‡^: p<0.05. PT_low_ *versus* PT_c_: ***: p<0.001. PT_low_ *versus* T_c_: ^††^: p<0.01; ^†††^: p<0.001.

**FIGURE 1:**
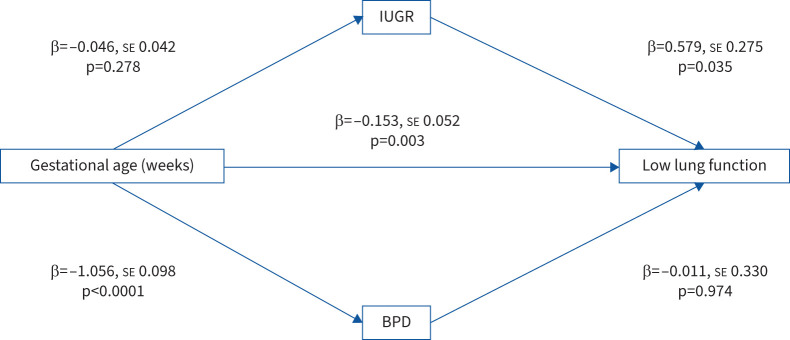
Mediation analyses of the relationship between gestation, intra-uterine growth restriction (IUGR) and bronchopulmonary dysplasia (BPD) with low lung function in preterm-born children.

We next identified which early-life factors were associated with low spirometry in the preterm group. Univariable analyses showed that gestation, IUGR and diagnosis of BPD were significantly associated with low FEV_1_ % pred, but sex, mode of delivery, maternal smoking and WIMD quintile were not (table 3). With multivariable logistic regression analyses, gestation (β= –0.153, se 0.051; p=0.003) and IUGR (OR 1.783, 95% CI 1.06–3.00; p=0.029) remained significantly associated with low FEV_1_ % pred, but BPD (OR 0.99, 95% CI 0.52–1.89; p=0.974) did not (table 4), thus suggesting that gestation and fetal growth restriction, but not BPD, are important determinants of future deficits in FEV_1_. We next assessed if mild (n=40) or moderate/severe BPD (n=68) was a better predictor of decreased spirometry. On univariable analyses, both mild (OR 2.016, 95% CI 1.023–3.971) and moderate/severe BPD (OR 2.080, 95% CI 1.215–3.561) were significantly associated with low lung function, but neither was in a multivariable model (mild OR 1.099, 95% CI 0.493–2.449; p=0.818 and moderate/severe OR 0.917, 95% CI 0.439–1.917; p=0.818) also including gestation and IUGR (supplementary tables S2 and S3). To further elucidate the relationships between the early-life factors and low lung function, we used mediation analyses (figure 1). Using mediation analyses, gestation was significantly associated with BPD, but not with IUGR, when the two preterm groups with and without low spirometry were compared. However, IUGR was significantly associated with low lung function in the preterm group, but BPD was not, thus consolidating the multivariable logistic regression results that gestation and IUGR were associated with low lung function in the preterm group.

**TABLE 3:** Univariable analysis of predictors of low lung function in the preterm-born population

	**β**	**se or OR (95% CI)**	**p-value**
**Covariates**			
Gestational age (weeks)	–0.153	0.036	0.000*
**Factors**			
IUGR (reference=No)	0.606	1.83 (1.10–3.06)	0.020*
BPD (reference=No)	0.721	2.06 (1.31–3.22)	0.002*
Family history of asthma^#^ (reference=No)	0.240	1.27 (0.86–1.88)	0.228
Sex (reference=Female)	–0.165	0.85 (0.58–1.24)	0.399
Antenatal smoking^#^ (reference=No)	−0.206	0.81 (0.43–1.53)	0.521
Postnatal smoking^#^ (reference=No)	−0.276	0.76 (0.42–1.37)	0.359
Caesarean section^#^ (reference=No)	−0.024	0.98 (0.66–1.44)	0.905
WIMD quintiles			
1 (most deprived)	–0.110	0.99 (0.54–1.80)	0.971
2	–0.181	0.84 (0.46–1.51)	0.550
3	0.242	1.27 (0.73–2.23)	0.397
4	–0.184	0.83 (0.47–1.48)	0.532
5 (least deprived)	Reference		

Covariates presented as β and se; β and OR (95% CI) presented for all factors. IUGR: intra-uterine growth restriction; BPD: bronchopulmonary dysplasia; WIMD: Welsh Index of Multiple Deprivation 2019. ^#^: missing cases: family history of asthma n=5, antenatal smoking n=12, postnatal smoking n=5, caesarean section n=2. *: p<0.05.

**TABLE 4:** Multivariable modelling for low lung function in the preterm-born population

	**β**	**se or OR (95% CI)**	**p-value**
**Covariates**			
Gestational age (weeks)	–0.153	0.051	0.003*
**Factors**			
IUGR (reference=No)	0.579	1.783 (1.06–3.00)	0.029*
BPD (reference=No)	–0.011	0.99 (0.52–1.89)	0.974

Covariates presented as β and se; β and OR (95% CI) presented for all factors. IUGR: intra-uterine growth restriction; BPD: bronchopulmonary dysplasia. *: p<0.05.

## Discussion

In this study, we investigated preterm-born children who had low lung function in childhood to determine which early-life factors could explain these deficits. Gestation and IUGR were strongly associated with the lung function deficits, but mild and moderate/severe BPD, although both were associated with low lung function in univariable analyses, were not following adjustments for gestation and IUGR. Mediation analyses showed that gestation led to an association with both low spirometry and development of BPD, and IUGR was independently associated with low spirometry, but BPD was not.

It is well established that preterm-born children who had BPD in infancy have decreased lung function [7, 8, 24], but it is increasingly recognised that BPD is not an optimal marker of future lung function decrements. It can be argued that a better definition of BPD [25, 26] can potentially improve this predictability, but any refined definition is unlikely to optimally identify lung disease in preterm-born children who did not have BPD in infancy, especially those born at ≥32 weeks of gestation who are less likely to develop BPD. Thus, in this study, since we and others have shown that children who are born late preterm are at risk of future lung dysfunction [3, 9, 10], we included children who were born at 33–34 weeks of gestation as well those born at ≤32 weeks of gestation who are traditionally considered to be at risk of developing BPD [6]. Indeed, although 38% (41 out of 108) of the BPD group had low spirometry, a significant proportion (23% (100 out of 436)) from those without BPD including those born late preterm also had low lung function. The results remained similar with 38% of the BPD group and 25% of the preterm controls having low lung function when we confined the analyses to those born at ≤32 weeks of gestation. The greater proportion in the BPD group was most likely due to the lower gestation observed in this group.

In order to explore factors that may be associated with respiratory disease in the future, we initially classified the preterm-born children into those with low and normal spirometry, basing the classification on a pragmatic approach for the research nurses to use a cut-off value of FEV_1_ 85% predicted that was close to the lower limit of normal for the group we studied to identify children who could join RHiNO, which includes a randomised controlled trial of inhalers. Although it is tempting to attribute the lower findings to the diagnosis of BPD, gestation was more influential for future spirometry deficits in multivariable logistic regression modelling, but the diagnosis of BPD was not. The data suggest that gestation and IUGR are strong determinants of low lung function in preterm-born children, thus suggesting that delivery at an early stage of lung development is most likely to be most associated with future lung dysfunction [27]. Traditionally, studies have compared lung function, including spirometry and impulse oscillometry, in preterm-born children with and without BPD, generally showing that BPD is associated with decreased lung function. Hurst *et al.* [7] showed decreased measures of spirometry in young adults born at <26 weeks of gestation with BPD when compared with those without or with term-born controls. In contrast, Manti *et al.* [28] did not show a significant difference in impulse oscillometry between preschool age children with and without BPD. Some studies have investigated early-life factors that could explain later lung function. In a longitudinal study, Levin *et al*. [29] reported that duration of mechanical ventilation and neonatal corticosteroid exposure in a multivariable model adjusting for BPD and gestation were associated with lower rate of rise in FEV_1_ % pred in those with BPD. In contrast, another study of 6-year-old children (n=88, including n=78 with BPD) reported that Nissen's fundoplication was associated with lower FEV_1_ [30]. Studies thus far have been limited by power, suggesting the need for larger studies such as ours.

*F*_ENO_ has previously not been shown to be increased in preterm groups who do or do not have BPD in infancy [31–33]. Therefore, it was interesting to note that *F*_ENO_ was increased in 23% of the PT_low_ group and only 9% and 11% in the PT_c_ and T_c_ groups, respectively, suggesting that at least some of the PT_low_ children are likely to have an inflammatory process occurring. Similarly, a greater proportion of the PT_low_ group responded to single administration of salbutamol (29% *versus* 7% *versus* 6% for the PT_low_, PT_c_ and T_c_ groups, respectively). Taken together, these data suggest that the PT_c_ group is similar to the T_c_ group and likely to have good respiratory health in the future. In contrast, the PT_low_ group appears to have some individuals who have high *F*_ENO_ and some who respond to bronchodilators, suggesting that different phenotypes [34] are likely to exist within this group, which requires further dissection.

The strengths of this study are the large number of prospectively and comprehensively studied preterm-born children together with term controls and the use of standardised methods by two highly trained research nurses. Weaknesses include fewer responses than we had anticipated. Differences noted especially for the deprivation score are unlikely to introduce any bias as the main aim was to study preterm-born children with low lung function to identify their associations with early-life factors, so the findings should remain robust and are clearly independent of response rates. In addition, the questionnaire data may be subject to parental recall bias. Thus, we have concentrated more on objective measures of spirometry as outcome measures.

In summary, we have shown that although moderate/severe BPD is associated with longer term lung function deficits, gestation and IUGR are better determinants of decreases of lung function. In addition, children with low lung function had increased *F*_ENO_ and greater bronchodilator responses when compared with preterm and term controls. By identifying children with low lung function, which we have termed “prematurity-associated lung disease”, we can better study the underlying mechanisms of why these children continue to have lung function decrements in the future and to optimise therapies [35].

## Supplementary material

10.1183/13993003.01766-2021.Supp1 **Please note:** supplementary material is not edited by the Editorial Office, and is uploaded as it has been supplied by the author.
Supplementary material ERJ-01766-2021.Supplement

## Shareable PDF

10.1183/13993003.01766-2021.Shareable1 This one-page PDF can be shared freely online.
Shareable PDF ERJ-01766-2021.Shareable
