# Supplementary material for: Association of early-life factors with prematurity-associated lung disease: prospective cohort study
Source: Eur Respir J. 2022 May 12;59(5):2101766. doi: 10.1183/13993003.01766-2021 (PMC9095942; doi:10.1183/13993003.01766-2021)
Supplement: Supplementary file 1 [file ERJ-01766-2021.Supplement.pdf]

Association of Early Life Factors and Prematurity-Associated Lung Disease: A Prospective Cohort Study

[1,2]Kylie Hart, [1,2]Michael Cousins, [1]W John Watkins, [1]Sarah J Kotecha, [3]A John Henderson**, [1,2]Sailesh Kotecha.

**This publication is dedicated to our expert collaborator, valued mentor, and very dear late friend.

[1]Department of Child Health, Cardiff University School of Medicine, Cardiff, United Kingdom.

[2]Neonatal Unit, Cardiff and Vale University Health Board, Cardiff, United Kingdom.

[3] MRC Integrative Epidemiology Unit, Population Health Sciences, Bristol Medical School, University of Bristol, Bristol, United Kingdom.

Online Figure S1: CONSORT diagram of children invited to join the study

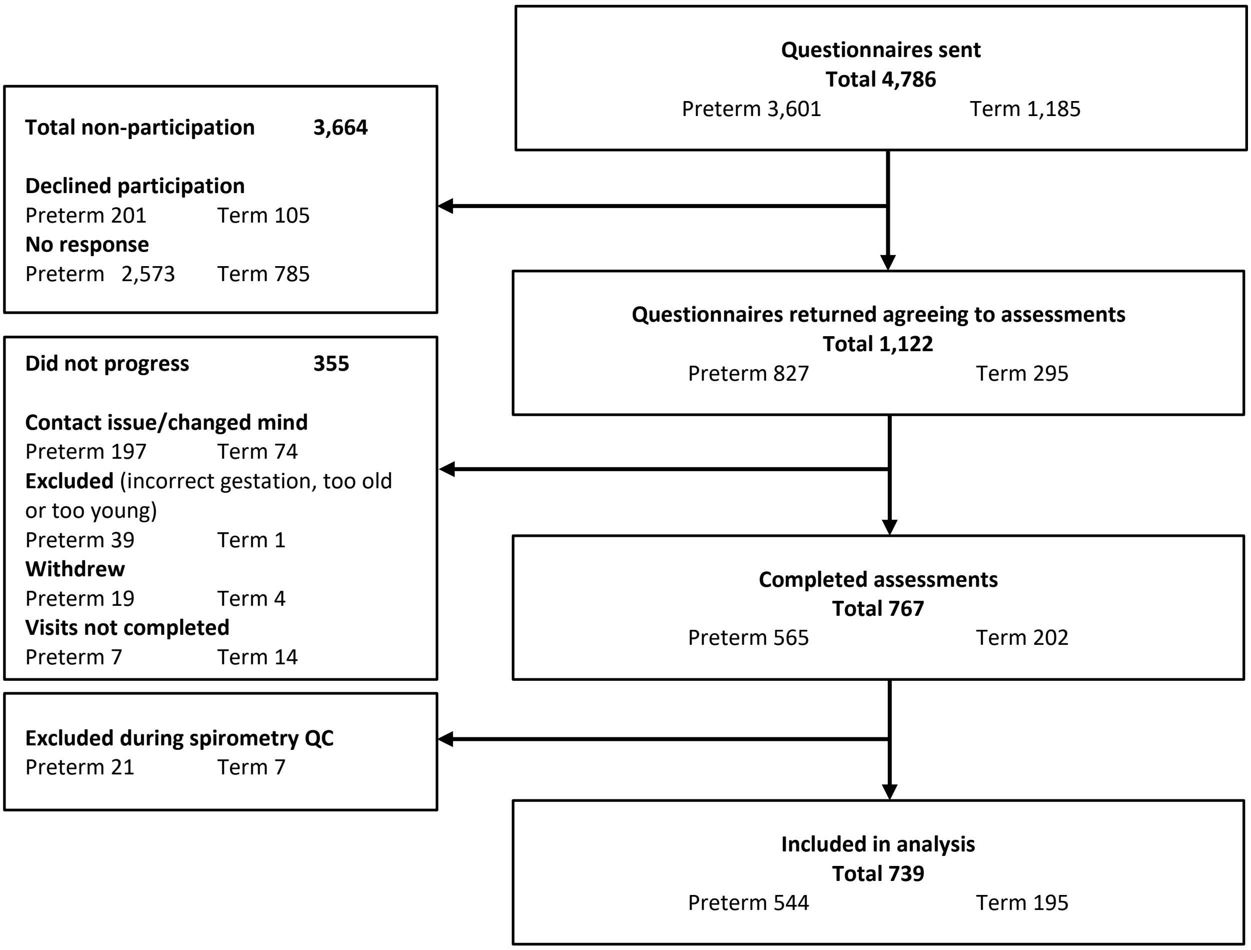

Online Table S1: Comparison between the responders and non-responders' groups

	Preterm		Terms	
	**Responders**	**Non-responders**	**Responders**	**Non-responders**
**Subjects (n)**	565	3036	202	983
Male	293/565 (52%)	1679/3036 (55%)	100/202 √√√ (50%)	513/983 (52%)
Gestational age (weeks) (mean SD)	31.0 (2.7) √√√	31.6 (2.6)	39.8 (1.2) √	39.6 (1.3)
Birthweight (g) (mean SD)	1703 (564) √√√	1828 (684)	3480 (479)	3443 (507)
WIMD 2019 (mean SD)	1062 (570) √√√	813 (583)	1192 (522) √√√	1025 (558)
1 – most deprived	91/563 (16%) √√√	850/2,731 (31%)	16/198 (8%)√√	161/981 (16%)
2	103/563 (18%)	615/2,731 (23%)	28/198 (14%)	183/981 (19%)
3	103/563 (18%)	420/2,731 (15%)	41/198 (21%)	202/981 (21%)
4	114/563 (20%)	362/2,731 (13%)	44/198 (22%)	204/981 (21%)
5 – least deprived	152/563 (27%)	484/2,731 (18%)	69/198 (35%)	231/981 (24%)

Abbreviation: WIMD – Welsh Index of Multiple Deprivation.

Significance √p<0.05, √√p,0.01, √√√p<0.001 comparing the responders and non-responders in the preterm or the term groups.

Number for responders are those who consented for a visit but before any QC.

Online Table S2: Univariable analysis of BPD as a predictor of low lung function in the preterm-born population

**Univariate analyses**			
**Factors**	**Beta**	**Odds ratio (95% confidence interval)**	**Significance**
No BPD (Ref)	-	**1**	**-**
Mild BPD	0.701	**2.016 (1.023, 3.971)**	**0.043**
Moderate/Severe BPD	0.732	**2.080 (1.215, 3.561)**	**0.008**
Beta value and Odds Ratios with 95%CI			

Online Table S3: Multivariable modelling for low lung function in the preterm-born population

**Model 1**			
**Covariates**	**Beta**	**Standard error**	**Significance**
Gestational age	-0.155	0.052	**0.003**
**Factors**	**Beta**	**Odds ratio (95% confidence interval)**	**Significance**
IUGR (ref = No)	0.591	**1.806 (1.069, 3.049)**	**0.027**
Mild BPD (ref = No BPD)	0.094	1.099 (0.493, 2.449)	0.818
Moderate/Severe BPD	-0.087	0.917 (0.439, 1.917)	0.818
Covariates presented as Beta value and SE. Beta value and Odds Ratios with 95%CI presented for all factors.			
