# Supplementary material for: Association of early-life factors with prematurity-associated lung disease: prospective cohort study
Source: Eur Respir J. 2022 May 12;59(5):2101766. doi: 10.1183/13993003.01766-2021 (PMC9095942; doi:10.1183/13993003.01766-2021)
Supplement: Supplementary file 2 [file ERJ-01766-2021.Shareable.pdf]

EUROPEAN RESPIRATORY JOURNAL
ORIGINAL RESEARCH ARTICLE

SHAREABLE PDF

Association of early-life factors with prematurity-associated lung disease: prospective cohort study

Kylie Hart[1,2], Michael Cousins[1,2], W. John Watkins[1], Sarah J. Kotecha[1], A. John Henderson[3] and Sailesh Kotecha[1,2]

[1]Dept of Child Health, Cardiff University School of Medicine, Cardiff, UK. [2]Neonatal Unit, Cardiff and Vale University Health Board, Cardiff, UK. [3]MRC Integrative Epidemiology Unit, Population Health Sciences, Bristol Medical School, University of Bristol, Bristol, UK.

Corresponding author: Sailesh Kotecha (KotechaS@cardiff.ac.uk)

Check for updates

Shareable abstract (@ERSpublications)
Although traditionally bronchopulmonary dysplasia is thought to be associated with longer term lung function deficits, this study shows that gestation and fetal growth restriction are better predictors of lung function deficits in prematurely born children https://bit.ly/3obSdSz

Cite this article as: Hart K, Cousins M, Watkins WJ, *et al*. Association of early-life factors with prematurity-associated lung disease: prospective cohort study. *Eur Respir J* 2022; 59: 2101766 [DOI: 10.1183/13993003.01766-2021].

This single-page version can be shared freely online.

Copyright ©The authors 2022.

This version is distributed under the terms of the Creative Commons Attribution Licence 4.0.

Received: 22 June 2021
Accepted: 16 Sept 2021

Abstract

Background Although bronchopulmonary dysplasia (BPD) is associated with lung function deficits in childhood, many who develop BPD have normal lung function in childhood and many without BPD, including those born at 33–34 weeks of gestation, have lung dysfunction in childhood. Since the predictability of BPD for future lung deficits is increasingly doubted, we prospectively recruited preterm-born children to identify early-life factors associated with lung function deficits after preterm birth.

Methods From 767 children aged 7–12 years who had their respiratory symptoms assessed, and had spirometry before and after a bronchodilator in our Respiratory Health Outcomes in Neonates (RHiNO) study, 739 (544 preterm-born at ⩽34 weeks of gestation and 195 term-born) had satisfactory lung function. Data were analysed using multivariable logistic regression and mediation.

Results When preterm-born children were classified according to their lung function, low lung function (prematurity-associated lung disease (PLD)) was associated with BPD, gestation and intra-uterine growth restriction (IUGR) on univariable logistic regression analyses. However, on multivariable logistic regression analyses, gestation (β=–0.153, SE 0.051; p=0.003) and IUGR (OR 1.783, 95% CI 1.06–3.00; p=0.029) remained significantly associated with later deficits of lung function, but BPD (OR 0.99, 95% CI 0.52–1.89; p=0.974) did not. Mediation analyses confirmed these results.

Conclusions Although traditionally BPD has been associated with low lung function in later life, the data show that gestation and IUGR are significantly associated with PLD in childhood, but BPD is not. By identifying children with PLD, we can better understand the underlying mechanisms and develop optimal therapies.
